# Supplementary material for: Topical Protease Inhibitor Increases Tumor-Free and Overall Survival in CD4-Depleted Mouse Model of Anal Cancer
Source: Viruses. 2024 Sep 5;16(9):1421. doi: 10.3390/v16091421 (PMC11436184; doi:10.3390/v16091421)

Supplemental Figure S1. Tumor-free survival when lymphoma mice removed. The changes that were noted in tumor-free survival when the lymphoma mice were removed included a change in the significance p value DMBA only CD4 vs. SQV+DMBA CD4 is now p=0.0644 (ns)

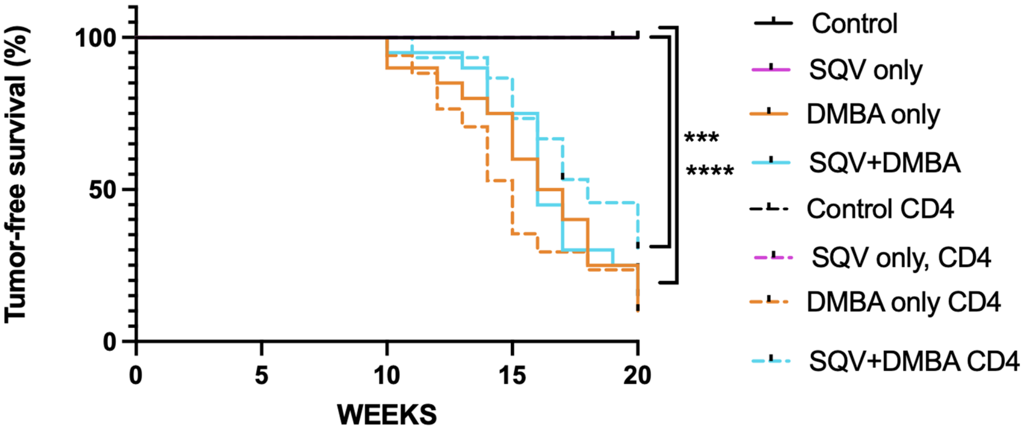

Supplemental Figure S2. Overall survival when lymphoma mice removed. The changes that were noted in overall survival when the lymphoma mice were removed included a change in the significance p-value DMBA only CD4 vs SQV+DMBA CD4 is now p=0.0185 (*) instead of p=0.00441 (**).

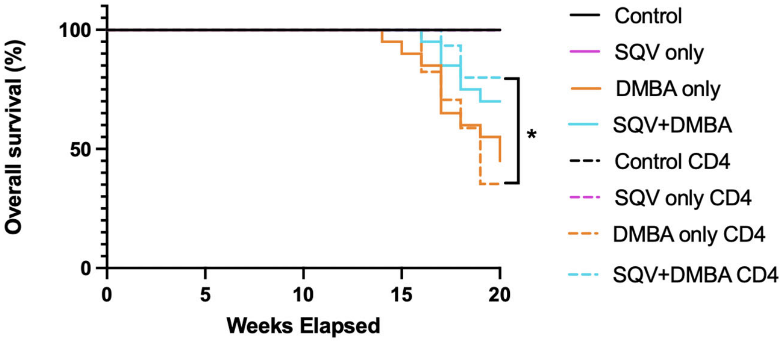

Supplemental Figure S3. Tumor volumes over time with lymphoma mice removed. No changes that were noted in tumor-free survival when the lymphoma mice were removed.

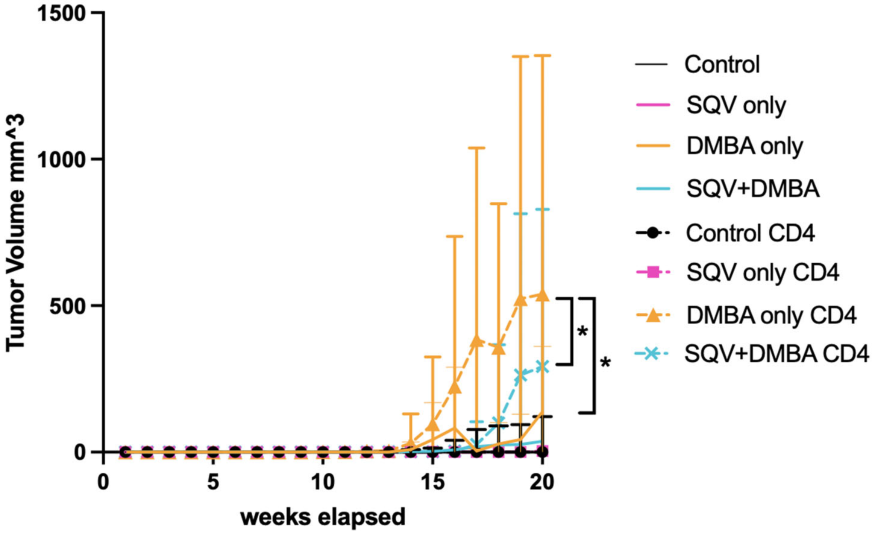

Supplemental Figure S4. Histology with lymphoma mice removed. For the control group there were 20 mice: 5 had low-grade dysplasia and 15 had high-grade dysplasia. For the SQV only group there were 20 mice: 10 had normal histology and 10 had low grade dysplasia. For the DMBA only group there were 20 mice and they all had cancer. For the SQV+DMBA group there were 20 mice: 2 had low-grade dysplasia, 9 had high-grade dysplasia, and 9 had SqCC. For the control CD4 depleted group there were 13 mice: 2 had normal histology, 1 had low-grade dysplasia, 8 had high-grade dysplasia, and 2 had SqCC. For the SQV only CD4 depleted there were 14 mice: 2 had normal histology, 2 had low-grade dysplasia, 8 had high-grade dysplasia, and 2 had SqCC. For the DMBA only CD4 depleted mice there were 16 mice: 8 had high-grade dysplasia and 8 had SqCC. For the SQV+DMBA CD4 depleted there were 15 mice: 8 with high-grade dysplasia and 7 with SqCC.

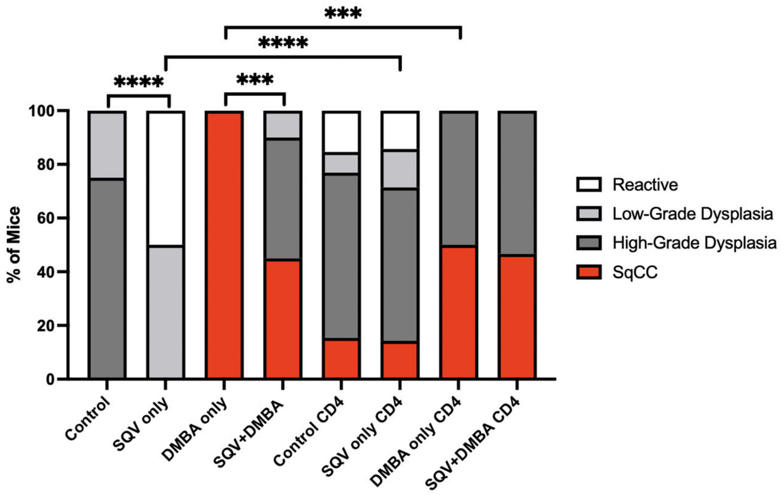

Supplement: Supplementary file 1 [file viruses-16-01421-s001.zip › viruses-3036921-supplementary.pdf]
